# Environmental impact of high-value gold scrap recycling

**DOI:** 10.1007/s11367-020-01809-6

**Published:** 2020-08-25

**Authors:** Benjamin Fritz, Carin Aichele, Mario Schmidt

**Affiliations:** 1 Institute for Industrial Ecology, Pforzheim University, Tiefenbronner Str. 65, 75175 Pforzheim, Germany; 2 Faculty of Sustainability, Leuphana University Luneburg, Universitatsallee 1, 21335 Luneburg, Germany

**Keywords:** Gold recycling, Gold refining, Gold mining, Life cycle assessment, Environmental impact, Aqua regia

## Abstract

**Purpose:**

The gold routes satisfying the global gold supply are mining (74%), recycling of high-value gold (23%), and electronic scraps (3%). Besides its applications in the investment, jewelry, and industrial sector, gold also has a bad image. The gold production in industrial as well as artisanal and small-scale mines creates negative impacts such as resource depletion, extensive chemical use, toxic emissions, high energy consumption, and social concerns that are of great importance. On the other hand, almost all gold is recycled and has historically always been. In common life cycle assessment (LCA) databases, there is no data on recycling of high-value gold available. This article attempts to answer the question what the ecological benefits of this recycling are.

**Method:**

In this study, we were able to collect process data on the most commonly used high-value gold scrap recycling process, the aqua regia method, from several state-of-the-art German refineries. With this data, life cycle inventories were created and a life cycle model was produced to finally generate life cycle impacts of high-value gold scrap recycling.

**Results:**

This study contains the corresponding inventories and thus enables other interested parties to use these processes for their own LCA studies. The results show that high-value gold scrap recycling has a considerably lower environmental impact than electronic gold scrap recycling and mining. For example, high-value gold scrap recycling in Germany results in a cumulative energy demand (CED) of 820 MJ and a global warming potential (GWP) of 53 kg-CO_2_-Eq. per kg gold. In comparison, common datasets indicate CED and GWP levels of nearly 8 GJ and 1 t-CO_2_-Eq. per kg gold, respectively, for electronic scrap recycling and levels of 240 GJ and 16 t-CO_2_-Eq. per kg gold, respectively, for mining.

**Conclusion:**

The results show that buying gold from precious metal recycling facilities with high technological standards and a reliable origin of the recycling material is about 300 times better than primary production.

**Electronic supplementary material:**

The online version of this article (10.1007/s11367-020-01809-6) contains supplementary material, which is available to authorized users.

## Introduction

Gold is used in many different products, from luxury accessories and securely guarded bars to tiny amounts in electronic goods. The gold entering our market comes either from mining or from recycling. The total gold supply in 2018 was 4670 tons, of which 23% was attributed to the refining of gold-containing scraps such as jewelry or coins, 3% came from the recycling of waste electrical and electronic equipment (WEEE), and the rest was newly mined gold (Hewitt et al. 2015; GFMS 2019).

It is well known that most precious metals have major environmental impacts since large pits or deep shafts must be dug in the ground to extract relatively small amounts of the desired metals. The ore contents in gold mining range from only half a gram per ton of ore, for example, in the artisanal and small-scale mining (ASM) in Brazil to several tens of grams per ton of ore in the large industrial mines in Canada or Australia. Furthermore, chemicals such as cyanide or mercury are used for extraction. The widely quoted study by Earthworks (Septoff 2004) stated, for example, that a wedding ring produces approximately 20 tons of toxic waste. On the other hand, gold is almost perfectly recycled because of its value and precious metal properties. However, what are the ecological benefits of this process, and could it be that we are doing wrong to the material gold if we lump all production routes together?

The newly mined gold can further be divided into the two categories of primary and secondary deposits. Primary deposits are ores that formed during the original mineralization periods, as opposed to secondary deposits that are a result of alteration or weathering (Pohl 2005; Renner et al. 2012). Primary deposits are mined either using open pits or underground mining, while secondary deposits are mainly mined from water bodies with dredges or by washing old riverbeds using hoses (hydraulic mining) (Priester and Hentschel 1992; McQueen 2005). Today, secondary deposits are almost solely exploited by ASM, in contrast to large-scale commercial mines.

What all mines have in common is that so-called dore bars are first produced, which, in addition to gold, contain other elements such as silver or mercury. Dore bars are usually shipped to internationally recognized refineries, which cast them on site and produce high-quality (99.99% purity) bars (Eibl 2008). However, certain refineries differ from others in that these refineries refine or recycle only scraps but not dore bars.

**Fig. 1:**
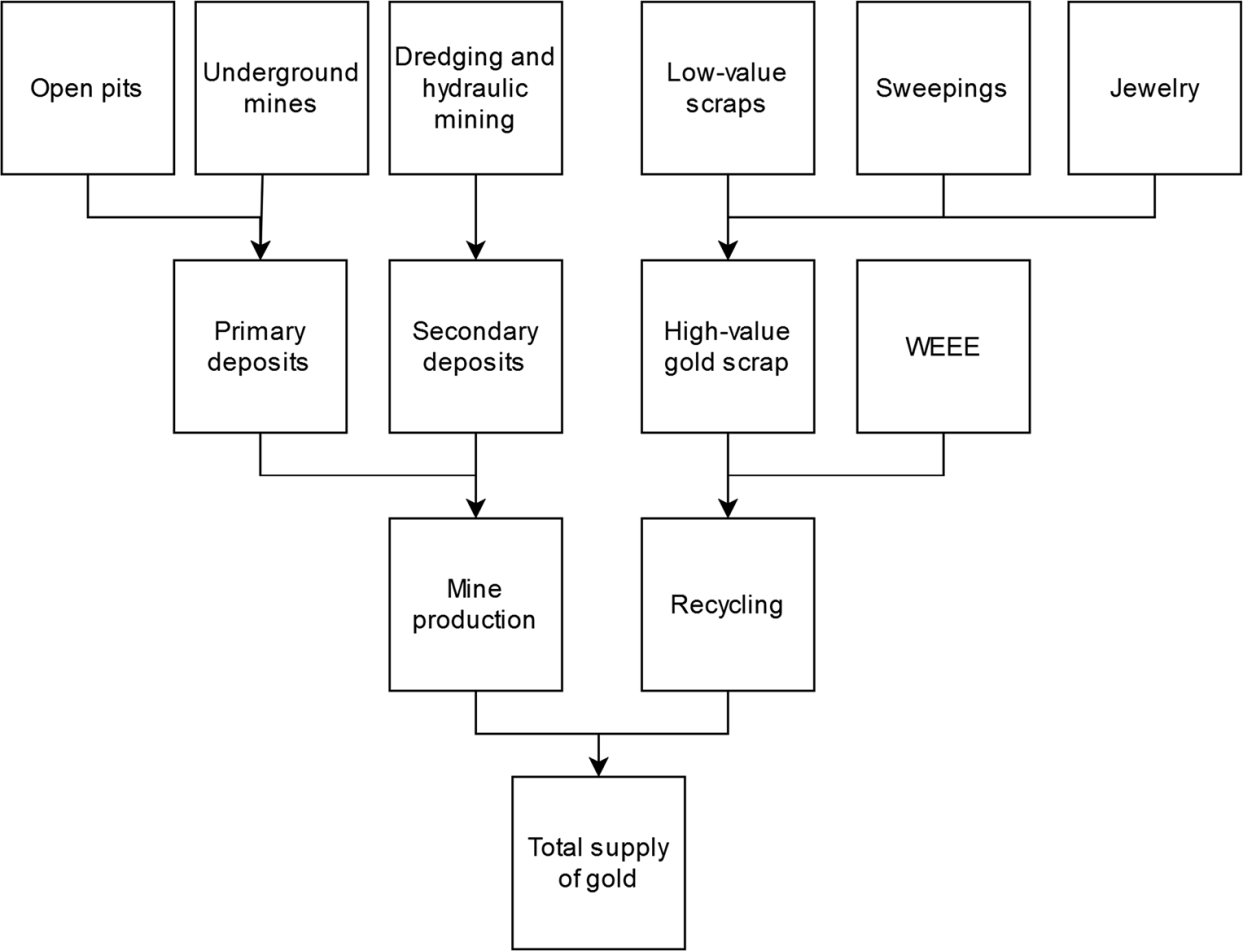
Compositions of the different gold production routes of the market

These precious metal recycling facilities are the focus of this study, and their scrap input is further divided into three groups: high- and low-value gold scraps and sweepings. High-value gold scraps mainly consist of jewelry with some coins and bars with a high gold content. Low-value scraps are versatile in their occurrences but mainly originate from the automobile and electronic industries (World Gold Council 2018). Sweeping waste is mainly waste from jewelers like residues from polishing, clothes, floor sweepings, etc. (Ferrini 1998; Renner et al. 2012). The composition of the different gold production routes can be seen in Fig. 1.

There are several different gold refining processes. The process used depends mainly on the size of the refinery and the type of input material (George 2015). Certain processes, such as *Miller chlorination* or *Wohlwill electrolysis*, are better suited to refine primary materials from mines such as the aforementioned dore gold on a large scale (Corti 2002). Other processes, such as *aqua regia*, are better suited to refine secondary high-value gold scraps (Chmielewski et al. 1997; Sum 1991). More precisely, the aqua regia process is recommended for refining high-value (> 75% Au), non-dore scraps, since it is the fastest, simplest, and most robust process (Adams 2016).

Interestingly, in addition to its decorative use as jewelry, gold serves a dual function. First, in tiny amounts, gold satisfies various industrial needs, and second, in bars, coins, and sometimes even jewelry, gold is used as a safe investment. For gold producers, the profit generated by gold, as for any other product, is made up of the turnover less the costs. In contrast to other products, there is no need to worry about the sales and customer acquisition markets. This characteristic is the main incentive for the simple gold prospectors in the ASM sector. Metaphorically speaking, digging for gold is like digging for money. The aboveground gold stocks comprise 48% jewelry, 31% investment applications, and 21% industrial and other uses (World Gold Council 2018). With all the gold in banks and in private possession, strictly speaking, gold should not be a critical or scarce material, at least not for industrial purposes.

An established analysis method of the environmental impacts along the life cycle of products is life cycle assessment (LCA). In their study on the environmental impacts of smartphones by Ercan et al. (2016) using the LCA database ecoinvent for upstream chain activities, it was reported that gold contributes to five impact categories at 50% or higher (the largest contribution was to ecotoxicity at 60%). Similar results were reported by O’Connell and Stutz (2010) in their analysis of the product carbon footprint of a Dell laptop, stating that the main contributing component to the carbon footprint is the random-access memory (RAM), where the gold pins account for a significant share of the carbon footprint. In an extensive LCA dataset, the research by Nuss and Eckelman (2014) on the environmental impacts of the cradle-to-gate processes of 63 metals showed that gold is among the most polluting elements on a kilogram basis. As a result, the environmental impact of gold is present in product LCA studies to the extent that the ecological image of gold has also attracted the attention of public media.

On the other hand, at the global and annual scales, gold has comparatively low environmental impacts because of its rather low production volumes in contrast to those of steel or iron, for example (Nuss and Eckelman 2014; World Gold Council 2018).

**Fig. 2:**
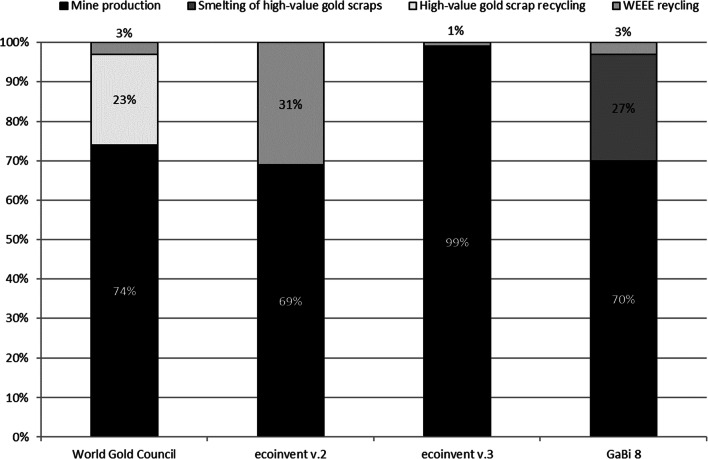
Representation of the gold route shares in the different LCA datasets (private communication)

Since the environmental impacts of even the smallest quantities of gold are so high in product LCAs, it is important to be able to represent the market activities in the underlying LCA databases as realistically as possible. The current database situation is as follows: the gold production datasets in ecoinvent v.3.5 contain assumptions and aggregations from one mining site to another. Mine tailing data, to give one example, is extrapolated on the basis of the mass of gold production volume from one open gold-silver mining pit in Papua New Guinea to eight other open and underground mine sites around the world. The ASM sector is still not included in any datasets today. To understand the situation in the LCA databases focusing on recycling, we recall the gold route ratios mentioned at the very beginning of this chapter with 74% of the gold coming from mines, 23% attributed to high-value gold scrap recycling, and 3% originating from WEEE recycling (Classen et al. 2009). In the ecoinvent v.2.2 market datasets, which are intended to cover the average global production of gold, a share of 30% is used for secondary production, and since only WEEE recycling data are available, it is assumed that the 30% share can be completely attributed to gold from WEEE recycling (Classen et al. 2009). Since WEEE recycling involves a large number of different materials with different compositions but a low content of valuable metals, high-value gold scrap recycling is therefore a less elaborate process than WEEE recycling (World Gold Council 2018). In the ecoinvent dataset v.3.4, this shortcoming is corrected by omitting the mass fraction of high-value scraps, resulting in a 99% gold share from mining and a 1% gold share from recycling, which is also not representative of the real situation. In the GaBi database (PE International 2019), the high-value gold recycling route is represented with a share of 27%, but the route is represented by a simple smelting process without refining processes, which probably does not reflect reality since refineries have more complex metallurgical processes than just smelting. An overview of how the different LCA databases represent the gold supplying routes, contrasted to what is known from market statistics, is shown in Fig. 2.

On the other hand, it is supposed that of the approximately 190,000 tons of gold mined until today, the amount of gold historically lost is approximately 2 to 15% (Butterman and Amey 1996; George 2015; GFMS 2019). This supposition is made because gold has the special characteristics that it has always been valuable, is resistant to corrosion and oxidation, and was therefore always recycled or rather reused. Gold could be considered a kind of exemplary case study for the concept of the circular economy (CE), which started forty centuries ago. However, at the same time, this study fits well in the discussion about the limits of the CE because even in the case of a high-value and noble metal, it is not possible to completely close the loop.

Until now, it has been difficult to obtain data on gold recycling processes, as the gold market as a whole tends to keep information intended for the public discreet. This study helps to better understand how effective the recycling of gold scraps into fine gold is. The study could even serve as a prime example in Germany, compared with the prominent and well-publicized stories of the WEEE recycling sites in the developing countries such as the Agbogbloshie market in Accra City, Ghana (Asante et al. 2012; UNEP et al. 2019; Ongondo et al. 2011). Furthermore, we are aiming to develop life cycle inventories for this specific process route. The question we are trying to answer for the first time is the following: how can we close the data gap in terms of the gold from precious metal recycling facilities to raise the integrity of the market activities involved in the gold supply within the LCA context?

## Methodology

To finally examine and close the LCA data gap of this missing 23% share of the total gold supply from recycling, an extensive study was conducted on the processes commonly used for the recycling of gold scraps and how these processes work. Subsequently, for the first time primary data were gathered for the prior detected processes from a number of state-of-the-art precious metal recycling facilities in Pforzheim with a production volume of approximately 50 tons of gold per year. As the refineries under investigation are located in Germany, there are many laws and regulations that employers must follow to ensure the well-being and fair and equal treatment of employees. For reasons of confidentiality, the facilities must remain anonymous, but these facilities represent rather a best practice case for the gold scrap recycling in Germany and thus in highly industrialized countries.

To use the collected data for environmental assessments such as LCA, certain general specifications were agreed upon. The system under investigation, or in other words the foreground system, ranges from the preparation of the refinery input materials to the product output of 1 kg of 99.99% fine gold granulate. The 1 kg of 99.99% also represents the function unit (FU). The system boundary is a cradle-to-gate system. This boundary was determined because in recycling, a cut-off system model is typically used, and the other phases, such as collection or transport, are negligible (1% of the total GWP) compared with chemical processing due to the small shipment quantities (see supplement a).

Metals often occur as by-products in multi-output processes, e.g., as ore bodies in mining or as scraps in recycling containing multiple valuable fractions. As a result, the environmental impacts of these processes must be distributed among the value-adding precious metals. The most common method for solving this problem, also recommended by the DIN ISO EN 14044, is allocation by mass or monetary value. Allocation by mass means that the metal with the largest quantity is assigned the highest environmental impacts and vice versa. Allocation by monetary value means that the metal with the highest value in the process (mass times the market value) will have the highest impacts (Bruijn et al. 2004). In LCA studies on metals, the prevailing opinion is that mines are only operated because their products have a high monetary value (Tuusjärvi et al. 2012). Often mines are closed when gold prices make mining unprofitable and reopened later when the gold price rises. In the current COVID-19 pandemic, we observe this phenomenon especially in ASM, as the price of gold is high and the price of oil is low. Nevertheless, for the inventory of the in- and output flows of the high-value gold recycling process in this study, allocation was performed using both mass and economic factors. For a more detailed explanation of the allocation method, see Supplementary material b.

**Fig. 3:**
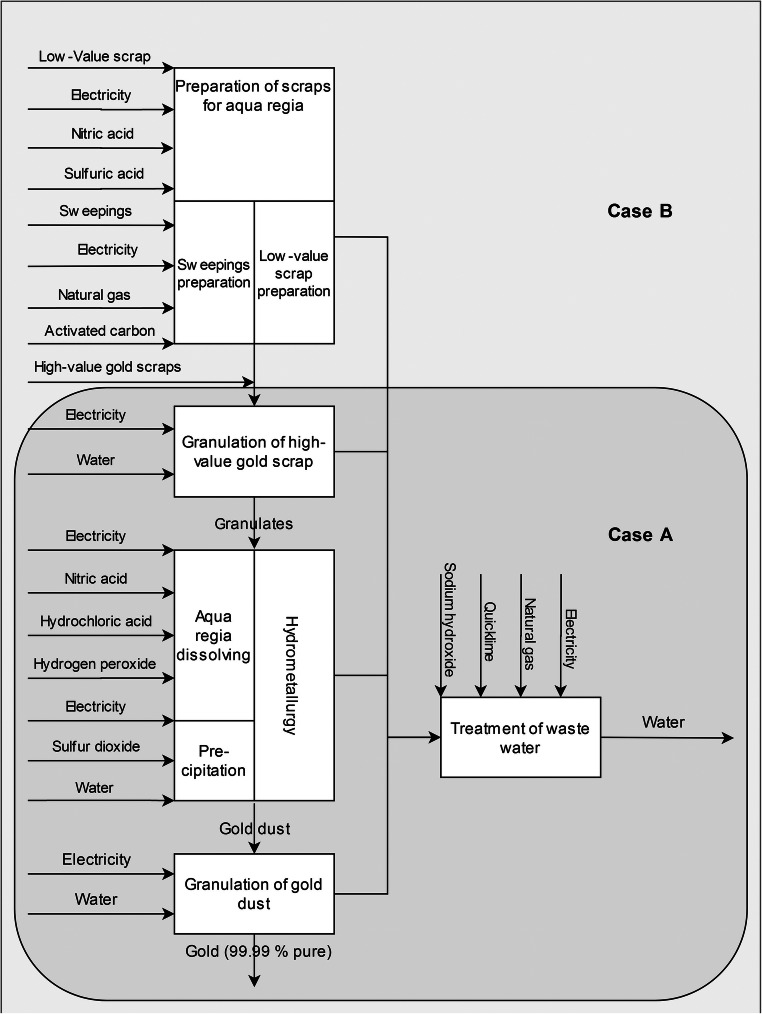
Schematic diagram of the process of gold refining

Different refineries recycle different qualities of scrap depending on economic and technological decisions. In practice, this condition means that there are various processes upstream of the aqua regia process to bring the different input scraps to concentrations suitable for the aqua regia process. These processes are mainly electro- and pyrometallurgic. To make this as meaningful as possible in this study, we will distinguish two different cases. Case A will represent the ceteris paribus case for the hydrometallurgical treatment of high-grade gold scrap using the aqua regia process. Case B is an extension of Case A with the abovementioned electro- and pyrometallurgic upstream processes. The ratios in case B are based on the mean values of the primary company data as we witnessed during the on-site visits in this study. Their quantities of low- and high-value scraps and sweepings are given in Table 1. It is important to note that the inventory for the preparation of low-value scraps condenses several different processes for the different scrap input qualities for reasons of simplicity. The two cases A and B are shown in the schematic diagram of the aqua regia process as used in this article in Fig. 3.

**Table 1:** Inventory for different processes with mean values gathered from various refineries in Germany (values rounded)

Input			Output		
Process: low-value scrap preparation (amount per kg lv)					
	By mass	By monetary value		By mass	By monetary value
Low-value scraps	250 kg	250 kg	Wastewater, internal	0.3 kg	14 kg
Electricity	12 MJ	270 MJ	Gold-enriched low-value scraps (lv)	1 kg	1 kg
Nitric acid	58 g	1.1 kg			
Sulfuric acid	37 g	4.6 kg			
Process: sweepings incineration (per kg sweepings)					
Sweepings		1.9 kg	Carbon monoxide, fossil		0.38 g
Electricity		11 MJ	Carbon dioxide, fossil		6.3 kg
Natural gas		2.5 m^3^	Hydrogen chloride		0.14 g
Activated carbon		17 g	Nitrogen oxides		12 g
			Particulates, > 2.5 μm and < 10 μm		61 mg
			Sulfur dioxide		0.20 g
			Sweepings, ashes (sa)		1 kg
Process: granulation of scraps, prepared (per kg scrap)					
High-value scraps		0.44 kg	Scraps, prepared and granulated for the aqua regia process (sg)		1 kg
Gold-enriched low-value scraps		50 g	Wastewater, internal		13 kg
Sweepings, ashes		0.51 kg			
Electricity		0.45 MJ			
Tap water		13 kg			
Process: aqua regia (per kg Au)					
Scraps, prepared and granulated for the aqua regia process		3.3 kg	Silver, in silver chloride		0.45 kg
Electricity		45 MJ	Palladium, in solution		59 g
Hydrochloric acid		3.4 kg	Platinum, in solution		44 g
Hydrogen peroxide		6.4 kg	Non-valuable fraction		1.7 kg
Nitric acid		0.7 kg	Wastewater, internal		24 kg
Sulfur dioxide		0.93 kg	Gold dust		1 kg
Tap water		13 kg			
Process: granulation of gold dust for sale (per kg Au)					
Gold dust		1 kg	Gold		1 kg
Electricity		1.5 MJ	Wastewater, internal		13 kg
Tap water		13 kg			
Process: wastewater (per kg wastewater)					
Wastewater, internal		1.1 kg	Carbon dioxide		38 g
Sodium hydroxide		5.6 g	Wastewater, river disposal (ww)		1 kg
Quicklime		45 g	Hydroxide sludge		180 g
Natural gas		20 l			
Electricity		0.39 MJ

The first step is to prepare the input for hydrometallurgical refining with aqua regia to guarantee a mixture ratio that is suitable for the aqua regia treatment. The preparation of low-value scraps involves many different types of electrolysis processes, depending on the different qualities of the input material and the technologies available in the refinery. The preparation of sweepings entails the incineration of the inputs, which burns off all the organic material. In this step, activated carbon is added to the flue gas stream to reduce the emissions (e.g., carbon mono- and dioxide, hydrogen chloride, nitrogen oxides, sulfur dioxide, or mercury). The input of high-value scraps mainly consisting of jewelry and coins is not subject to any further preparation in addition to sampling. Second, a mixture ratio of the three inputs that is suitable for solvation in aqua regia is defined. This mixture is then smelted and sent through small holes of approximately half a centimeter in diameter into water to create small granulates that are easy to dissolve in acid later. The smelting process is electrically heated. The granulated scraps are then dissolved in aqua regia, an acid consisting of a mixture of one part concentrated nitric acid and three parts concentrated hydrochloric acid. The following reaction (Eq. 1) summarizes the process.

1 $$ Au+{HNO}_3+4 HCl\to HAu{Cl}_4+ NO+2{H}_2O $$

In this step, electricity is mainly used for keeping the temperature at approximately 90 °C and for peripheral components such as pumps and stirrers. This step forms silver chloride that can be gravitationally separated from the solution. Now the gold has to be precipitated from the solution. One common method is the addition of sulfur dioxide (Adams 2016). Gold is precipitated with sulfur dioxide by the following reaction (Eq. 2):

2 $$ 2{HAuCl}_4+3{SO}_2+6{H}_2O\to 2 Au+8 HCl+3{H}_2{SO}_4 $$

Next, the denser gold fraction, occurring as fine gold dust, can be gravitationally separated from the solution. The fine gold dust is smelted and regranulated for sale. The remaining solution still contains small quantities of platinum and palladium, which are separated in an additional process step. Since this step is not necessary for the refining of the functional unit of 1 kg of gold*,* no further data on this process step are presented. The remaining chemical solution, together with the other wastewater flows (e.g., the electrolytes from the preparation of low-value scraps), is treated with sodium hydroxide and quicklime for neutralizing the pH value in a central wastewater unit. During this process, hydroxide flakes containing various metals are formed. These flakes are filtered out to form the so-called hydroxide sludge. The wet sludge is then dried with natural gas to reduce its weight and volume for disposal.

The processes related to the aqua regia processes as used in this article (Fig. 3) are then modeled in the LCA software Umberto NXT (ifu Hamburg GmbH). This software was chosen because of its effectiveness in modeling multi-output processes, handling different allocation rules, as well as cost calculations and its good options for visualizing the results, e.g., Sankey diagrams, which are useful when working together with industry partners. The gate-to-gate model was extended to a cradle-to-gate model by using the background processes from the ecoinvent v.3.5 database. Wherever possible, attempts were made to use market datasets for Germany [DE] or countries with a similar technological development level, since the primary gate-to-gate data originate from German factories. Sodium hydroxide is the only exception, and as there are no other data, a global (GLO) process had to be chosen. The processes used are summarized in Supplementary Section c in Table iii. Additionally, for one process, the incineration of sweepings elementary exchanges with their associated environmental impacts from the ecoinvent v.3.5 materials in the category *non-urban air or from high stacks* was used (see Table 1), since we had primary data from emission measurements that fitted well to the materials available in ecoinvent on hand.

To determine the relevant impact categories for the environmental impact assessment related to this inventory, a literature review of 12 different LCA studies was conducted in which gold was assessed either as the main focus of study or as a by-product (e.g., WEEE recycling). The impact categories used in these studies were then aligned with the 14 characterization factors recommended by Hauschild et al. (2013) to generate a consolidated list of characterization factors. In other words, combining the relevant environmental impacts gained from the literature research with the characterization factors recommended by Hauschild et al. (2013) (and thus by the EU initiative ILCD), a comprehensive list of relevant impact assessment methods for the material gold is obtained. A detailed list of all the assessed LCA studies can be found in Supplementary material c. Since five of the articles reviewed used the cumulative energy demand (CED), this parameter will be included in this study, although it was not included in the Hauschild et al. (2013) study.

## Results

### Gate-to-gate inventory

The data we have collected is based on real, on-site measurements and quantities. In a few cases, consumption quantities could not be allocated exactly to processes. In these cases, reasonable estimates and allocations were made in coordination with the personnel and then validated using literature values or stoichiometric calculations.

For the preparation of low-value gold scraps, data from only one refinery are available. The low-value scraps undergo a different electrolysis process depending on the precious metal contents. In practice, the gold content of the scrap inputs is highly concentrated with each electrolytic separation step of the base metals. Within this preparation, we therefore have several processes that indeed separate or produce other valuable metals at a considerably high purity level, e.g., copper or silver. As we encounter multi-output processes here, we need to apply allocations (see Section 2). Because only one refinery is processing these scraps and because the refinery data are confidential, we agreed to publish only the data allocated for the product output of the prepared low-value scraps (lv). All the processes were modeled in Umberto with the amounts and monetary values of the different in- and outputs. Subsequently, these processes were combined into one process by using allocations according to the ecoinvent system models v.3 (by monetary value) and v.2 (by mass) to bear exactly the same environmental impacts as the disaggregated processes. The aggregated inventory for the electrolytic preparation of the different low-value scraps is provided in Table 1. The material designation “wastewater, internal” means that this wastewater enters the internal wastewater treatment unit and is not directly piped into the municipal sewage system. This process is part of case B, and the mean gold concentration in the prepared low-value scraps is 18%.

For the incineration of sweepings, it was possible to gather the emissions measurements for this process from one company. For the sake of simplicity, substances occurring in trace amounts in the emissions data were disregarded if these substances did not have extraordinarily high-impact factors in one of the impact categories considered in this study. These data were then extrapolated correspondingly on the basis of the mass of the scrap inputs for the average value of all refineries, which can be found in Table 1. This process is part of the aforementioned case B, and the average concentrations of valuable metals in the ash of the incinerated sweepings (si) are as follows: Au, 0.69%; Ag, 12%; Pd, 0.010%, and Pd, 0.08%.

Table 1 shows the data of the granulation of all scrap inputs, namely, the high-value scraps (e.g., jewelry and coins), the low-value scraps subjected to various electrolysis processes, and the incinerated sweepings. This inventory is particularly interesting as it shows the mean ratio of the companies’ scrap input flows to the following hydrometallurgical refining step with aqua regia. This ratio is also the ratio used to distinguish between cases A and B introduced in Section 2. The average concentrations of the high-value scrap inputs are as follows: Au, 61%; Ag, 14%; Pd, 4.0%; and Pt, 2.9%.

The inventory data for the hydrometallurgical refining process with aqua regia and the subsequent granulation of gold dust can be found in Table 1.

The companies that provided the primary data had one central wastewater cleaning unit to fulfill the legal regulations for wastewater disposal in Germany. Because these data were not process-specific and since it was concluded from the internal discussions that water is not internally circulated, it was assumed that the amount of wastewater is equal to the input mass of auxiliary materials (e.g., chemicals) plus the non-valuable scrap fraction (see Table 1). The primary process data for this process were scaled down to the *per-amount-of-wastewater-basis* (ww) and then extrapolated using the aforementioned wastewater amount. The carbon dioxide emissions associated with the combustion of natural gas to dry the hydroxide sludge were stoichiometrically calculated, resulting in 1.90-kg carbon dioxide per m^3^ of gas (at 288.15 K and 101.325 kPa).

### Cradle-to-gate inventory

Next, the materials of the process inventories, or, so to speak, the foreground system of the LCA model (see Section 3.1), have been extended by the aggregated background processes (see Table iii of the Supplementary material) of ecoinvent to create a cradle-to-gate inventory for the production of 1-kg gold for case B. This condensed table contains in its rows the required energy by energy source and some resulting emissions for the respective aggregated background processes in the columns. The materials required for multiple processes, such as electricity, are not duplicated incorrectly. For example, the production of 3.4-kg hydrochloric acid requires electricity, the generation of which in turn requires coal. This coal is then only represented in the hydrochloric acid production column and not incorrectly entered again in the electricity production column, which only contains the amount of coal needed to produce 140 MJ of electricity. For the scrap preparation, the values were determined on the basis of the allocation by monetary value (see Table 1), as provided in Table 2.

**Table 2:** Cradle-to-gate inventory for the aqua regia process

*	Unit	Electricity, medium voltage (140 MJ)	Hydrochloric acid (3.4 kg)	Hydrogen peroxide (6.4 kg)	Nitric acid (0.88 kg)	Sulfur dioxide (0.93 kg)	Tap water (69 kg)	Sulfuric acid (0.76 kg)	Natural gas (5.7 m^3^)	Activated carbon (0.03 kg)	Sodium hydroxide (0.42 k g)	Quicklime (3.4 kg)
Energy carriers												
Coal	g	18	0.90	1.8	0.05	0.125	0.01	0.02	0.04	0.13	0.24	0.02
Crude oil	g	220	170	690	90	137	0.72	42	6.7	3.8	23	320
Gas	l	860	270	1500	160	260	0.77	80	5600	14	44	15
Uranium	mg	180	22	38	0.74	3.4	0.32	0.27	0.31	0.46	2.2	2.1
Hydropower	MJ	8.7	2.5	4.2	0.12	0.38	0.07	0.05	0.03	0.05	0.50	1.23
Biomass	MJ	11	1.6	2.7	0.12	0.22	0.01	0.10	0.02	0.03	0.14	0.04
Geothermal	kJ	70	71	120	2.4	10	0.15	1.00	0.50	1.44	16	0.80
Solar	kJ	2.7	0.63	4.27	0.12	0.07	0.01	0.02	0.02	0.05	0.07	0.40
Wind	MJ	17	0.90	1.5	0.03	0.14	0.004	0.01	0.03	0.02	0.10	0.02
Emissions												
Carbon monoxide	g	19	2.7	6.5	0.42	0.51	0.11	0.16	2.4	0.25	0.50	16
Carbon dioxide	kg	26	2.1	7.3	0.56	0.31	0.01	0.06	0.80	0.21	0.53	3.8
Methane	g	40	5.0	28	0.87	1.8	0.03	0.51	1.8	0.62	1.4	0.46
Nitrogen oxides	g	26	5.0	11	5.0	0.82	0.03	0.55	1.1	0.60	1.4	1.9
Sulfur dioxide	g	26	11	18	3.3	45	0.03	4.9	17	1.0	1.6	1.9
Mercury	mg	9.2	0.72	1.3	0.04	0.07	0.01	0.01	0.03	0.04	0.11	0.02

### Life cycle impacts

Extending the cradle-to-gate model to include the characterization factors provides the impact assessment which can be used to analyze some of the environmental impacts of the gold refining process in Germany.

**Fig. 4:**
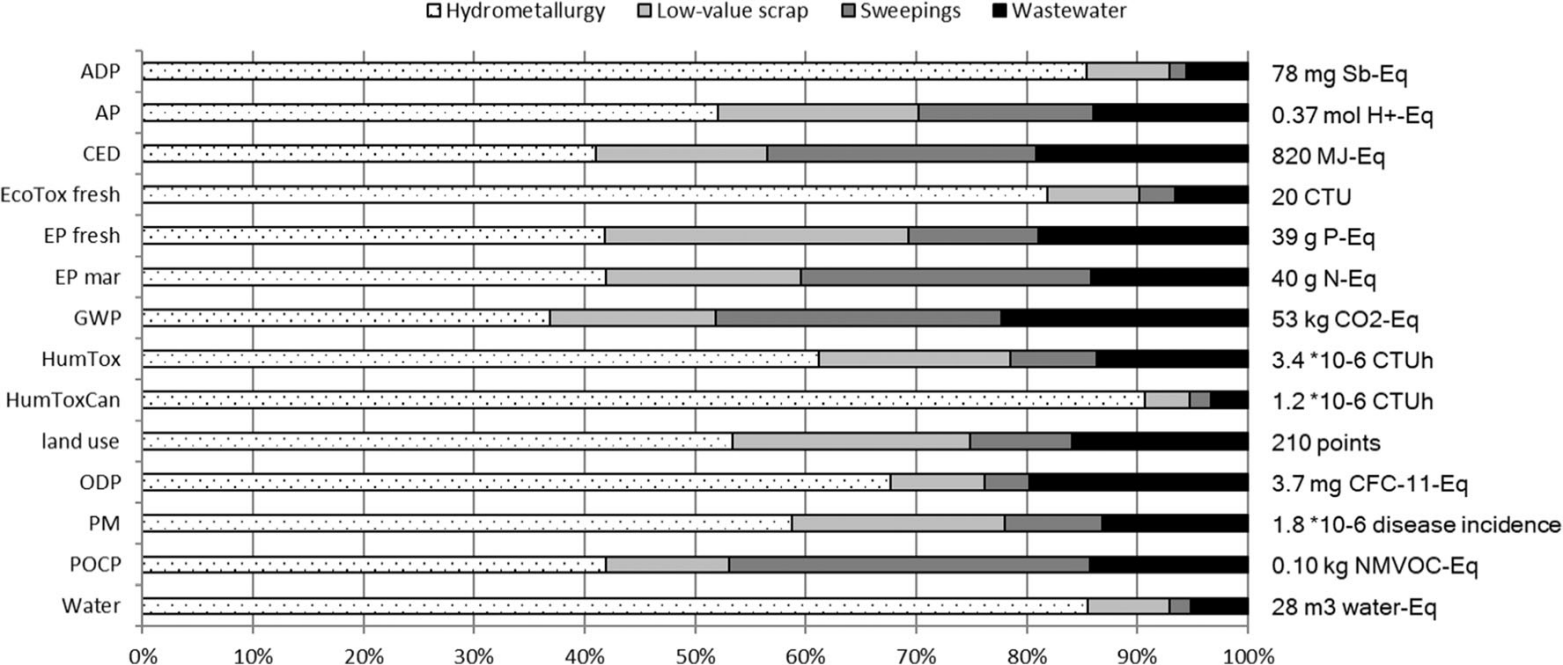
Environmental impacts of gold refining in Germany allocated by the monetary value

The graph in Fig. 4 shows the environmental impacts for 1-kg Au from the process route of case B for all the relevant impact categories mentioned in Section 2. The figure only shows the results allocated by the monetary value in order not to complicate the graph unnecessarily and since the environmental impacts in this method are higher, we are on the safe side with the conservative results. This approach is a common practice in the LCA field of precious metals. Further information on the abbreviations of the impact categories and their associated units can be found in the Supplementary material Section c. What stands out in the chart is that in all of the impact categories, the hydrometallurgy has the biggest impact and only in five categories its contribution to the overall impact is less than 50%.

Allocation by the monetary value gives far higher results than allocation by the mass because gold often occurs in very small quantities but is very valuable compared with metals such as copper or silver, which occur in rather large quantities. A more detailed analysis shows that a large part of the different results comes from the preparation of low-value scraps. This phenomenon is not surprising since the underlying metallurgical processes produce large quantities of low-value metals such as copper and silver (see Fig. i under Section b of the supplement).

**Fig. 5:**
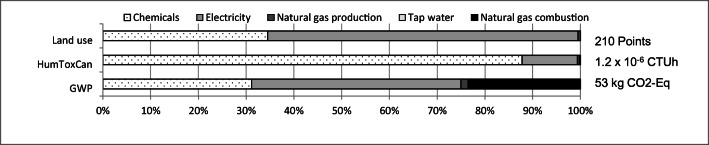
Impacts of the different material types on exemplary impact categories allocated by the monetary value

Figure 5 compares the total impact results of the three impact categories land use, HumToxCan, and GWP of the gate-to-gate inventories of case B (see Section 3.1), divided into the five different material types chemicals, electricity, natural gas production, and combustion as well as tap water per 1 kg Au. It is apparent from this figure that the chemicals and electricity are the highest contributing materials. What is striking about the chart is the very high contribution of chemicals to the human toxicity and thus to cancer effects. The contribution of the emissions measurement data from the incineration of sweeping to the impact categories HumToxCan or to land use is insignificant.

**Fig. 6:**
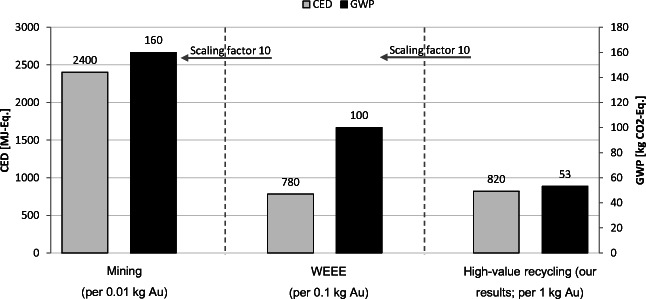
Comparison of the different gold routes from ecoinvent with this study’s new data on the recycling of gold scraps. Note that the values have been reduced by a factor of 10 for WEEE recycling and by 100 for mining

A comparison of the impact results of gold scrap recycling obtained in the context of this study as presented in Fig. 4, with the abovementioned ecoinvent v.3.5 LCA datasets on the gold from WEEE recycling and mining, is shown in Fig. 6. For this analysis, the model with allocation by the monetary value was chosen because, on the one hand, this method is the allocation method used in the ecoinvent v.3.5 datasets, and on the other hand, this model results in worse impacts, and with a conservative evaluation we do not run the risk of optimistically representing the study results. It is important to note that the values in this figure have been *reduced by a factor of 10 for WEEE* recycling and by a *factor of 100 for mining* to improve the graphical presentation. The results, as shown in the chart below, indicate that the findings of this study for gold from recycling of high-value scrap have a significant lower cumulative energy demand and global warming potential.

## Conclusion

The starting point of this study was that there were no LCA data on the recycling of gold scraps in any database and a possibility of closing this data gap in cooperation with refineries willing to help by providing primary data. Refineries usually tend to be rather reserved on such information. The real impact of these data on the overall picture became clearer during the study. A notable finding from the literature review was the 23% share of the gold coming from high-value gold scrap recycling. For the existing LCA datasets, this phenomenon immediately meant a 23% lower gold production from deep shafts and large holes associated with well-known environmental damage. Even more astonishing seems to be the fact that in Germany, almost all the processed gold is from gold scrap recycling. None of the refineries interviewed during this study accept dore gold. The only gold produced in Germany that comes directly from mining is found in by-products, e.g., copper concentrates. Although there might be a low probability of misdeclared gold scraps that are actually smelted dore gold smuggled into Germany and laundered in pawnshops, there is strong evidence that the amount is almost zero (Gronwald et al. 2019). The results of this study only apply to gold scrap that is not a primary material or new scrap and therefore only to refineries that only recycle input material from reputable sources. In addition, the refineries analyzed in the work are state-of-the-art precious metal recycling facilities with modern machinery and process flows as well as good waste management systems. These assumptions could be quite different in other countries. To solve the problem of nontransparent supply chains, technology such as blockchain could be used to increase the transparency of the gold origin.

In researching the state of knowledge of LCA concerning gold, the findings that underline the relevance of this work were the major environmental impacts that very small gold amounts today have on products such as smartphones or laptops. The results or the extension of the differences between the environmental impacts of gold scrap recycling with aqua regia and the literature results of previous studies are very positive (see Fig. 6 and Fig. ii in Supplement Section c).

Furthermore, it is interesting to note that more attention is paid to WEEE recycling than to high-value scrap recycling although quantitatively it is the minor fraction of gold recycling and also has worse environmental impacts. As this study is limited to Germany and electricity has a large influence on the overall results for 1 kg of gold from precious metal recycling, the spatial differences in the electricity markets of different countries and thus the spatial differences in the recycling of gold scraps play a significant role. For the end consumer seeking to purchase environmentally friendly gold, the results of the present study mean that the purchase of gold from precious metal recycling facilities in Germany is a good choice as its environmental impact is significantly lower than on world markets.

Nevertheless, there is still a need for further research in the field of LCA and the underlying LCA databases concerning gold. More primary data on the recycling routes in more countries are needed. The data collected in the framework of this study are exclusively from refineries in Germany that reflect well the best available technology. The ecoinvent data on gold from WEEE recycling are based on one recycling plant in Sweden. However, there are also data-related shortcomings in the field of industrial mining. For example, the datasets in ecoinvent v.3.5 include various estimates and extrapolations among the different mining sites. For tailings in particular, new studies have to be conducted to obtain more reliable primary data. Even though few details on the GaBi database are available, both the ecoinvent and GaBi datasets on primary gold do have one thing in common, namely, that the data almost exclusively rely on corporate reports. Therefore, according to Classen et al. (2009), “it must be assumed that the environmental impacts of gold mining are rather underestimated.” Another shortcoming that all databases have in common is caused by the missing data on the primary gold from ASM. To date, three studies have been identified with LCA data on the gold from ASM in Peru and the Philippines (Cenia et al. 2018; Kahhat et al. 2019; Valdivia and Ugaya 2011). To close this gap and include these data in common datasets, it is important to collect more data in different regions and, possibly even more difficult, to determine the market share of the gold from small-scale mining as accurately as possible.

## Electronic supplementary material

ESM 1 (DOCX 95 kb)
