# Supplementary material for: Environmental impact of high-value gold scrap recycling
Source: Int J Life Cycle Assess. 2020 Aug 25;25(10):1930–41. doi: 10.1007/s11367-020-01809-6 (PMC7445229; doi:10.1007/s11367-020-01809-6)
Supplement: Supplementary file 1 — (DOCX 95 kb) [file 11367_2020_1809_MOESM1_ESM.docx]

Supplementary Materials for the Article Environmental Impact of High-Value Gold Scrap Recycling

Authors: Benjamin Fritz ^a*^, Carin Aichele^a^, Mario Schmidt^a,b^

1. Institute for Industrial Ecology, Pforzheim University, Tiefenbronner Str. 65, 75175 Pforzheim, Germany
2. Faculty of Sustainability, Leuphana University Lüneburg, 21335 Lüneburg, Universitätsallee 1, Germany

*Institute for Industrial Ecology, Pforzheim University, Tiefenbronner Str. 65, 75175 Pforzheim, Germany, Benjamin.fritz@hs-pforzheim.de; Tel.: +49-723-128-6404, ORCID: 0000-0001-8072-6079)

## Transportation Worst Case Scenario

One of the precious metal recycling facilities has provided us with their shipping quantities and weights broken down by the different qualities of scrap. On average, one kg of gold had a gross shipping weight of 2.55 kg in year 2018. We assume that all shipments to the refinery have to be transported on average 300 km. We then further assume that this transport is done with a light commercial vehicle and not a semi-trailer or train. The ecoinvent data set *market for transport, freight, light commercial vehicle [Europe without Switzerland]* gives 1.87 kg-CO_2_-eq. / ton*km. Multiplying the 0.0025 tons with 300 km and 1.87 kg-CO_2_-eq. / ton*km equals to 1.4 kg-CO_2_-eq. / kg-Au. After allocation by mass to the gold content of the scrap shipments, this then makes 0.54 kg-CO_2_-eq. / kg-Au or a share of 1 % of the total greenhouse potential (53 kg-CO_2_-eq. / kg-Au). Even if allocated by monetary value, the result is still only 2.4% of the total greenhouse potential.

## Allocation

For the calculation of the allocation factors by mass the products (i) mass ($M_{i}$) has to be divided by the sum of all (n) the products masses. The calculation of the allocationfactor by mass is then as follows $\frac{M_{i}}{\sum_{i=1}^{4} M_{i}}$.

For the calculation of the allocation factors by monetary value the sum of the multiplication of price (P) with mass for all products is needed (for the underlying prices see table i). This sum will then be the denominator for the calculation of product whose factor needs to be calculated. The calculation of the allocation factor is then as follows $\frac{P_{i}*M_{i}}{\sum_{i=1}^{4} P_{i}*M_{i}}$.

Table i: Market prices (average prices from 2013 to 2018) for different commodities as used in environmental impact allocation

| Product | Price of metal in EUR |
| --- | --- |
| Gold | 34136.16 € |
| Silver | 496.37 € |
| Copper | 5.09 € |
| Palladium | 19939.14 € |
| Platinum | 31224.51 € |

The allocation factors used in this study can be found in table ii.

Table ii: Allocation factors used in this study (rounded)

| Product (Mass) | Allocation factor by mass | Allocation factor by monetary value |
| --- | --- | --- |
| Gold (1 kg) | 64 % | 92 % |
| Silver, in silver chloride (0.45 kg) | 3.8 % | 0.60 % |
| Palladium, in solution (59g) | 2.8 % | 3.2 % |
| Platinum, in solution (44g) | 29 % | 3.7 % |

A more detailed analysis of the total impact results for the global warming potential (GWP) and CED for the different allocation methods is shown in Figure i.


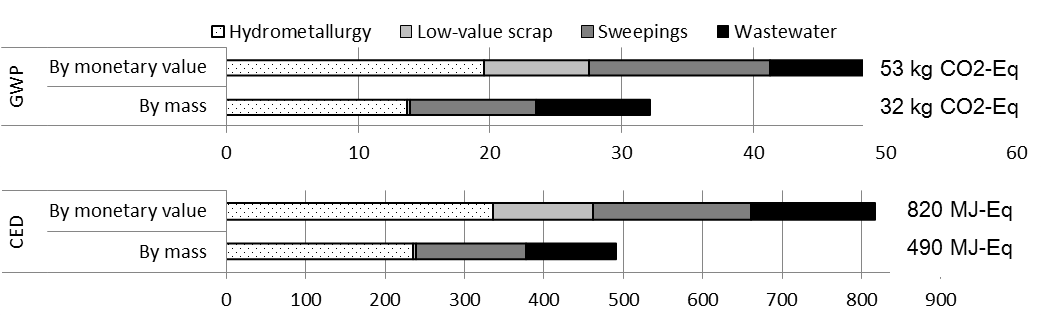


Figure i: Impact results representative of the GWP and CED levels allocated by mass vs. monetary value.

## Ecoinvent Processes

Table iii: Ecoinvent v.3.5 processes and their geographical locations are according to ecoinvent used to develop the cradle-to-gate inventory.

| **Process name** | **Geography** | **Purpose in this study** |
| --- | --- | --- |
| market for electricity, medium voltage | Germany [DE] | Temperature regulation, stirring and wastewater treatment |
| activated carbon production, granular from hard coal | Europe [RER] | Cleaning of flue gasses |
| market for tap water | Switzerland [CH] | Cooling and solidifying gold granulates |
| nitric acid production, product in 50% solution state | Europe [RER] | Dissolving gold in aqua regia |
| market for hydrochloric acid, without water, in 30% solution state | Europe [RER] | Dissolving gold in aqua regia |
| hydrogen peroxide production, product in 50% solution state | Europe [RER] | Dissolving gold in aqua regia |
| market for sulfuric acid | Europe [RER] | Electrolysis in low-value scrap preparation |
| market for quicklime, milled, loose | Switzerland [CH] | Adjusting the pH value |
| natural gas production | Germany [DE] | Smelting, incineration and drying |
| market for sodium hydroxide, without water, in 50% solution state | Global [GLO] | Cleaning of wastewater |
| treatment of wastewater from wafer fabrication, capacity 1.1E10l/year | Switzerland [CH] | Cleaning of wastewater |
| market for sulfur dioxide, liquid | Europe [RER] | Precipitating gold in aqua regia |

## Literature review

Table iv: List of common used impact categories in LCA studies subject to gold that match with (Hauschild et al., 2013) list of best characterization models

| Relevant Impact Categories | Acronym | Best characterization model | Unit | Espi (2009) | Kennecott (2016) | Thammaraksa (2017) | Pre (2012) | Nuss (2014) | Drielsma (2016) | Valdivia (2011) | Norgate (2012) | Mudd (2007) | TruCost (2017) | Li (2013) | Chen (2018) | Occurence in % |
| --- | --- | --- | --- | --- | --- | --- | --- | --- | --- | --- | --- | --- | --- | --- | --- | --- |
| Climate change | Global Warming Potential GWP | Baseline model of 100 years of the IPCC (Forster et al. 2007) | kgCO2-Eq | 1 | 1 | 1 |  | 1 |  | 1 | 1 | 1 | 1 | 1 | 1 | 83% |
| Acidification Potential | Acidification Potential[AP] | Accumulated exceedance (Seppälä et al. 2006; Posch et al. 2008) | mol H+-Eq | 1 | 1 |  |  | 1 |  |  | 1 |  | 1 | 1 | 1 | 58% |
| Resource depletion, water | [Water] | Model for water consumption as in the Swiss ecoscarcity (Frischknecht et al. 2008) | M3 water-Eq |  |  | 1 |  |  |  | 1 | 1 | 1 | 1 |  | 1 | 50% |
| Energy demand | Cumulative Energy Demand [CED] | Cumulative energy demand | MJ-Eq | 1 | 1 |  | 1 | 1 |  |  |  | 1 |  |  |  | 42% |
| Photochemical ozone formation | Photochemical ozone Creation Potential [POCP] | LOTOS-EUROS as applied in ReCiPe (Van Zelm et al. 2008) | Kg NMVOC-Eq |  | 1 | 1 |  |  |  |  | 1 |  |  | 1 | 1 | 42% |
| Land use | [points] | Model based on soil organic matter (SOM) (Milà i Canals et al. 2007) | points | 1 |  |  |  |  |  | 1 |  | 1 | 1 |  | 1 | 42% |
| Resource depletion, minerals and metals | Abiotic Depletion Potential [ADP | CML 2002 (Guinée et al. 2002) | kg Sb-Eq |  |  | 1 |  |  | 1 |  | 1 |  |  | 1 | 1 | 42% |
| Ozone depletion | Ozone depletion Potential[ODP] | Steady-state ODPs from the WMO assessment (Montzka and Fraser 1999) | kg CFC-11-Eq |  |  | 1 |  |  |  |  | 1 |  |  | 1 | 1 | 33% |
| Human toxicity, cancer effects | [HumToxCan] | USEtox model (Rosenbaum et al. 2008) | CTUh |  |  | 1 |  | 1 |  |  | 1 |  |  |  | 1 | 33% |
| Human toxicity, non-cancer effects | [HumTox] | USEtox model (Rosenbaum et al. 2008) | CTUh |  |  | 1 |  | 1 |  |  | 1 |  |  |  | 1 | 33% |
| Ecotoxicity, freshwater | [EcoTox fresh] | USEtox model, (Rosenbaum et al. 2008) | CTU |  |  | 1 |  |  |  |  | 1 |  | 1 |  | 1 | 33% |
| Particulate matter/respiratory inorganics | Particulate Matter [PM] | Compilation in Humbert (2009) based on Rabl and Spadaro (2004) and Greco et al. (2007) | Disease incidence |  |  | 1 |  |  |  |  | 1 |  |  |  | 1 | 25% |
| Eutrophication, aquatic / marine | Eutrophication Potential fresh- /marine water [EPfresh/ EPmar] | EUTREND model as implemented in ReCiPe (Struijs et al. 2009b) | kg P-Eq / kg N-Eq |  |  |  |  | 1 |  |  |  |  |  | 1 | 1 | 25% |

Espi JA, Morena SA (2010) The Scarcity-Abundance Relationship of Mineral Resource Introducing some Sustainability Aspects. DYNA 77:21–29; Kennecott Utah Copper (2007) Gold Environmental Profile - Life Cycle Assessment. http://www.kennecott.com/library/media/Gold%20Environmental%20Profile%202006.pdf. Accessed Nov 2017; Thammaraksa C, Wattanawan A, Prapaspongsa T (2017) Corporate environmental assessment of a large jewelry company. From a life cycle assessment to green industry. Journal of Cleaner Production 164:485–494. doi:10.1016/j.jclepro.2017.06.220; PRé (2012) LIfe cycle Assessment for a major jewelry manufacturer. https://www.pre-sustainability.com/download/Life-cycle-assessment-for-a-major-jewelry-manufacturer-A4.pdf. Accessed 06.2020; Nuss P, Eckelman MJ (2014) Life cycle assessment of metals: a scientific synthesis. PloS one 9(7):e101298. doi:10.1371/journal.pone.0101298; Drielsma JA, Russell-Vaccari AJ, Drnek T, Brady T, Weihed P, Mistry M, Simbor LP (2016) Mineral resources in life cycle impact assessment—defining the path forward. Int J Life Cycle Assess 21(1):85–105. doi:10.1007/s11367-015-0991-7; Valdivia SM, Ugaya CML (2011) Life Cycle Inventories of Gold Artisanal and Small‐Scale Mining Activities in Peru. Journal of Industrial Ecology 15(6):922–936. doi:10.1111/j.1530-9290.2011.00379.x; Norgate T, Haque N (2012) Using life cycle assessment to evaluate some environmental impacts of gold production. Journal of Cleaner Production 29-30:53–63. doi:10.1016/j.jclepro.2012.01.042; Mudd GM (2007) Global trends in gold mining. Towards quantifying environmental and resource sustainability. Resources Policy 32(1-2):42–56. doi:10.1016/j.resourpol.2007.05.002; Pandora, Trucost (2017) Material Sourcing Natural Capital Assessment and Net benefit Analysis. Trucost Assessment. https://pandoragroup.com/-/media/files/policies-and-statements/material-analysis-trucost.pdf. Accessed 06.2020; Chao Li et al. (2013) Life Cycle Assessment of different gold extraction processes. In: Energy Technology 2014 - Carbon Dioxide Management and Other Technologies. Wiley, J, Hoboken; Chen W, Geng Y, Hong J, Dong H, Cui X, Sun M, Zhang Q (2018) Life cycle assessment of gold production in China. Journal of Cleaner Production 179:143–150. doi:10.1016/j.jclepro.2018.01.114

In the following figure it is apparent that the variation of the literature data in the studies of mining is quite high (Figure ii)


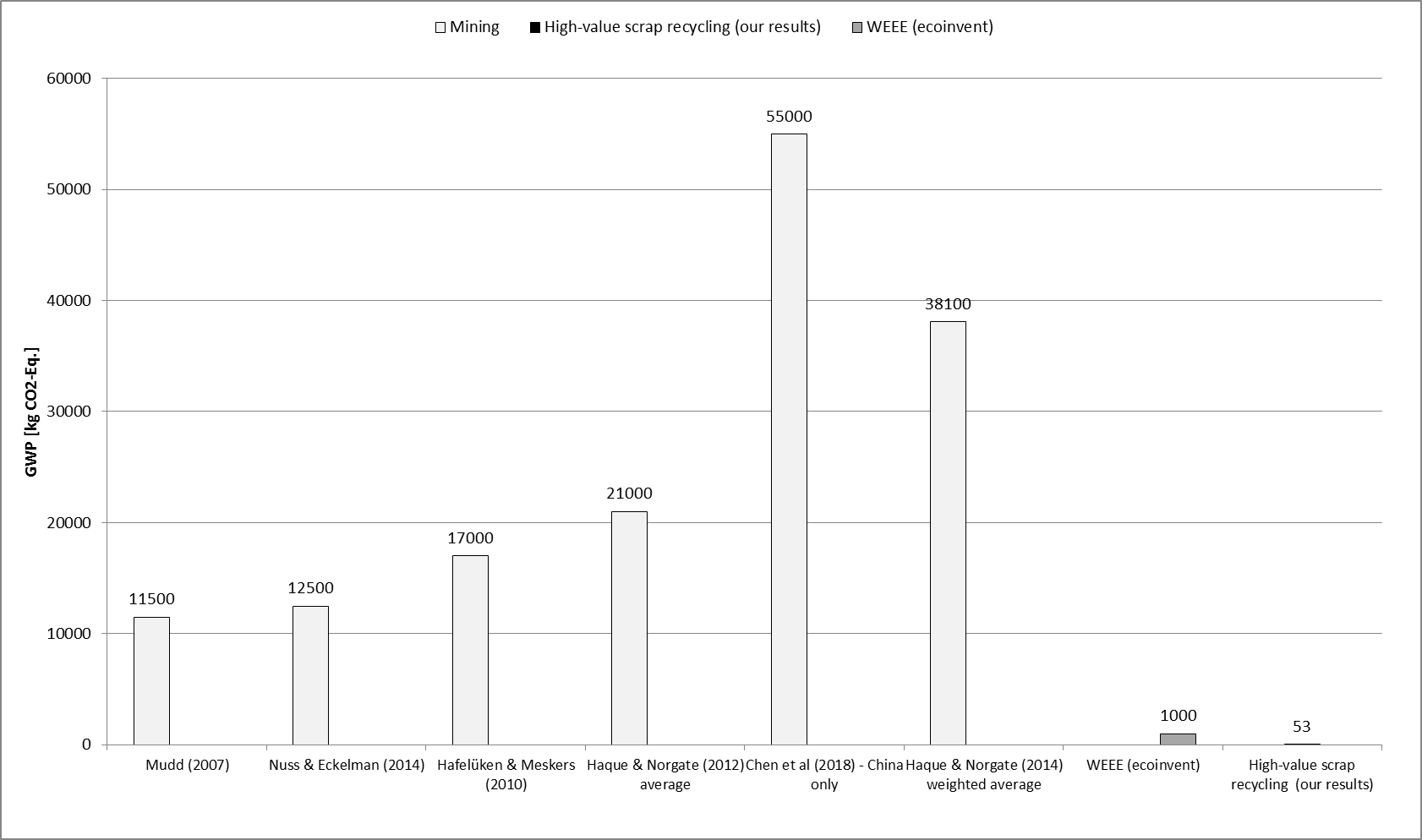


Figure ii: Comparison of the GWP results for mining from other studies with the ecoinvent’s data on WEEE recycling and this study’s new data on the recycling of gold scraps. The figure is modified from the World Gold Council (2018a).
